# A Novel Hybrid Approach for Risk Evaluation of Vehicle Failure Modes

**DOI:** 10.3390/s21020661

**Published:** 2021-01-19

**Authors:** Wencai Zhou, Zhaowen Qiu, Shun Tian, Yongtao Liu, Lang Wei, Reza Langari

**Affiliations:** 1 School of Automobile, Chang’an University, Xi’an 710064, China; 2017022004@chd.edu.cn (W.Z.); qzw@chd.edu.cn (Z.Q.); tianshun@chd.edu.cn (S.T.); qch_1@chd.edu.cn (L.W.); 2 Engineering Technology and Industrial Department, Texas A&M University, College Station, TX 77840, USA; rlangari@tamu.edu

**Keywords:** risk evaluation, vehicle failure mode, cost-based FMEA, FAHP, EFMULTIMOORA

## Abstract

This paper addresses the problem of evaluating vehicle failure modes efficiently during the driving process. Generally, the most critical factors for preventing risk in potential failure modes are identified by the experience of experts through the widely used failure mode and effect analysis (FMEA). However, it has previously been difficult to evaluate the vehicle failure mode with crisp values. In this paper, we propose a novel hybrid scheme based on a cost-based FMEA, fuzzy analytic hierarchy process (FAHP), and extended fuzzy multi-objective optimization by ratio analysis plus full multiplicative form (EFMULTIMOORA) to evaluate vehicle failure modes efficiently. Specifically, vehicle failure modes are first screened out by cost-based FMEA according to maintenance information, and then the weights of the three criteria of maintenance time (T), maintenance cost (C), and maintenance benefit (B) are calculated using FAHP and the rankings of failure modes are determined by EFMULTIMOORA. Different from existing schemes, the EFMULTIMOORA in our proposed hybrid scheme calculates the ranking of vehicle failure modes based on three new risk factors (T, C, and B) through fuzzy linguistic terms for order preference. Furthermore, the applicability of the proposed hybrid scheme is presented by conducting a case study involving vehicle failure modes of one common vehicle type (Hyundai), and a sensitivity analysis and comparisons are conducted to validate the effectiveness of the obtained results. In summary, our numerical analyses indicate that the proposed method can effectively help enterprises and researchers in the risk evaluation and the identification of critical vehicle failure modes.

## 1. Introduction

Recently, motivated by the explosive growth of the technical complexity of modern vehicles and their corresponding reliability [1], research on risk evaluation and safety assessment procedures has attracted much attention. Risk evaluation is regarded as an analytical method to assess quantitative and qualitative issues regarding various risk factors, and investigates potential results of possible problems on systems, designs, processes, services, etc. [2]. As one of the most promising risk evaluation methods, the failure mode and effects analysis (FMEA) method can identify and prioritize potential failure modes in fields whose risk has been assessed to figure out the causes and effects associated with such failure modes. In traditional FMEA, the rank of failure modes usually depends on the value of the risk priority number (RPN), which can be obtained by RPN=S×O×D, where S denotes the severity of the effect of the failure, O denotes the occurrence probability of the failure, and D denotes the detectability of the failure before its influence occurred [3,4]. In order to implement improvement measures to optimize vehicle design and maintenance effectively, the failure of obtaining a higher value of RPN should be analyzed as a priority. Therefore, after it was proposed in 1963 by NASA, FMEA was widely adopted to improve the safety and reliability in a wide variety of operational applications, such as in the aerospace, automobile and pharmaceutical industries [5,6,7]. Although conventional FMEA is regarded as an effective tool, it has certain drawbacks. For example, one of the major drawbacks is that the same RPN can be caused by different combinations of risk factors, which may increase the difficulty of risk control [8,9,10].

In addition, the value of RPN using traditional methods has been intensely condemned in previous research for its inaccuracy. Many studies [6,11,12] have presented solutions to overcome the drawbacks of FMEA. After Zadeh et al. [13] put forward the fuzzy set theory for the first time, a number of researchers [5,14,15] tried to describe the three risk factors in the FMEA method through fuzzy linguistic terms in conjunction with fuzzy “if–then” rules. Additionally, the conventional risk factors increased the difficulty of making an accurate judgment for experts who might not be familiar with FMEA. Ishii [16] defined cost as a risk factor to describe the conventional risk factors of S and D quantitatively. Indeed, a failure mode that is hard to detect would more easily increase the cost of maintenance. In the same vein, Khalilzadeh [17,18] has described a risk evaluation model with the risk factors of time, cost and profit to achieve the risk control for accidents in an industrial steel mill.

Among the various tools for risk evaluation [19,20,21], the multi-criteria decision making (MCDM) method, which can be used to explain experts’ preferences under multi-criteria environments both quantitatively and qualitatively, plays an important role due to its high effectiveness and feasibility [22,23]. As one of the most extensively developed MCDM methods, the comparison matrix is determined by utilizing fuzzy numbers instead of crisp numbers in the fuzzy analytic hierarchy process (FAHP) method. Laarhoven [24] first presented the FAHP to find weights for each criterion under a fuzzy environment. Gul [25] proposed a two-stage fuzzy multi-criteria method, where the weights of risk criteria were calculated through FAHP in the evaluation of a Turkish hospital. Currently, this method is always employed in conjunction with other risk evaluation methods for obtaining the weights [26,27]. As another useful risk evaluation tool, the multi-objective optimization by ratio analysis (MULTIMOORA) was purposed originally by Brauers and Zavadskas [28,29] and consisted of the ratio system, the reference point approach, and the full multiplicative form. In previous studies, this method has been conducted as a tool to perform risk evaluation in different fields [6]. For example, Gou [30] combined fuzzy linguistic language and MULTIMOORA to improve the effectiveness of conventional methods. Liu [31] also proposed a hybrid model according to this method as well as another decision-making methodology to evaluate risk priority in the health-care waste management system. Khalilzadeh [17] introduced a hybrid risk evaluation based on FMEA, AHP, and MULTIMOORA under a fuzzy environment for evaluating accidents in a factory, which obtained a better result than traditional risk criteria. In the above-mentioned studies, failure modes are usually classified by the opinions of experts or researchers, which may cause some confusion for experts who are not familiar with conventional criteria. For these reasons, a novel hybrid approach based on cost-based FMEA, FAHP, and the extended fuzzy MULTIMOORA (EFMULTIMOORA) method with new risk criteria, including maintenance time (T), maintenance cost (C), and maintenance benefit (B), is presented in this paper for evaluating the reliability of vehicles.

In this paper, we propose a three-stage scheme to evaluate the risk of vehicle failure modes. After classifying critical failure modes of vehicles using cost-based FMEA from maintenance information in the first stage, the weights of all risk criteria and the ranking of vehicle failure modes are obtained using FAHP and EFMULTIMOORA in the second and third stages, respectively. This therefore results in better accuracy in analyzing the judgments from the expert team as well as improving the feasibility and applicability of the risk evaluation method. Furthermore, it is easier for experts to judge the criteria used in EFMULTIMOORA and FAHP with new the risk criteria of T, C, and B, which can improve greatly with an improvement in vehicle reliability. In addition, a method for transforming triangular fuzzy numbers to crisp numbers is conducted in both previously proposed methods.

This paper is organized as follows. Section 2 describes the fuzzy set theory and a method for the defuzzification of triangular fuzzy numbers. Section 3 presents cost-based FMEA, FAHP, and EFMULTIMOORA methods. Section 3 also describes the proposed hybrid approach for evaluating vehicle failure modes. Section 4 details the proposed method using a case study of a Hyundai vehicle and elucidates its analysis results. Finally, conclusions and future work are discussed in Section 5.

## 2. Fuzzy Set Theory

### 2.1. Fundamental Theory of Fuzzy Set

**Figure 1:**
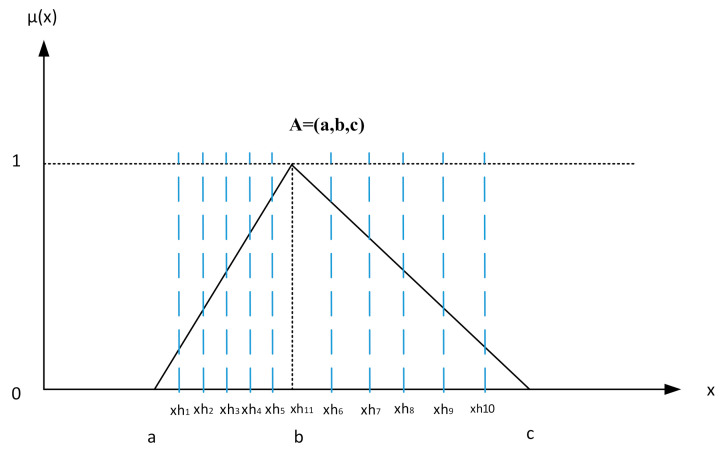
Fuzzy triangular number with 10-cut.

A fuzzy set is a collection of elements in a universe of information where the boundary of the set contained in the universe is ambiguous, vague, and otherwise fuzzy [32]. In this study, we consider fuzzy numbers as positive triangular fuzzy numbers, which are characterized by a membership function shown in Equation (1) and Figure 1 [33]. The arithmetic operations between two fuzzy numbers, such as $A=\left(\begin{array}{ccc} a, & b, & c \end{array}\right)$, $M=\left(\begin{array}{ccc} m, & n, & k \end{array}\right)$ and $r\in R^{+}$, are demonstrated as shown in Equations (2)–(6) [18].

(1) $$\mu \left(x\right)=\left\{\begin{array}{l} 0,x<a;\\ \frac{x-a}{b-a},a\leq x\leq b;\\ \frac{c-x}{c-b},b\leq x\leq c;\\ 0,x\geq c; \end{array}\right.$$

(2) $$A⊕M=\left[a+m,b+n,c+k\right]$$

(3) $$A\Theta M=\left[a-k,b-n,c-m\right]$$

(4) $$A⊗M≅\left(\begin{array}{ccc} am, & bn, & ck \end{array}\right)$$

(5) $$A⊙M≅\left[\begin{array}{ccc} \frac{a}{k}, & \frac{b}{n}, & \frac{c}{m} \end{array}\right]$$

(6) $$A\times r≅\left[ar\begin{array}{ccc} , & br, & cr \end{array}\right]$$

### 2.2. Defuzzification for Triangular Fuzzy Numbers

How to rank fuzzy numbers plays an important role in decision making [34]. Since all the vehicle failure modes are evaluated using fuzzy numbers, the comparison among these fuzzy numbers is indeed a comparison among alternatives. Usually it is necessary to transform the fuzzy numbers into crisp numbers to obtain better ranking results [35]. Such a transformation process can be defined as defuzzification. In this section, we introduce a method for defuzzification based on the method of α-cuts.

There are three possible conditions for the fuzzy number $A=\left(\begin{array}{ccc} a, & b, & c \end{array}\right)$. First, when (a=b=c), we can draw n vertical-cut lines with equal distances between a and b. Then we can get $xh_{i}$ and $yh_{i}$ using Equations (7) and (8), as follows. More details and proof about this defuzzification can be found in [14].

(7) $$xh_{i}=a+i\left(\frac{b-a}{n+1}\right),i=1,2,\ldots ,n$$

(8) $$yh_{i}=\left(\frac{1}{b-a}\right)*\left(xh_{i}-a\right),i=1,2,\ldots ,n$$

The same process is also performed for side bc, as shown in Equations (9) and (10), as follows.

(9) $$xh_{i}=a+i\left(\frac{c-b}{n+1}\right),i=n+1,n+2,\ldots ,2n$$

(10) $$yh_{i}=\left(\frac{-1}{c-b}\right)*\left(xh_{i}-b\right)+1,i=n+1,n+2,\ldots ,2n$$

where $xh_{2n+1}=b$, $yh_{2n+1}=1$, and $R_{A}$ is the crisp number correlated with A, which can be calculated as follows:

(11) $$R_{A}=\frac{1}{2n}\sum_{i=1}^{2n}\left(xh_{i}*yh_{i}^{0.01}\right)+b$$

When a=b<c and a<b=c, it can be obtained as follows:

(12) $$R_{A}=\frac{1}{2n}\sum_{i=1}^{2n}\left(xh_{i}*yh_{i}^{0.01}\right)+a$$

(13) $$R_{A}=\frac{1}{2n}\sum_{i=1}^{2n}\left(xh_{i}*yh_{i}^{0.01}\right)+b$$

## 3. Methodology

**Figure 2:**
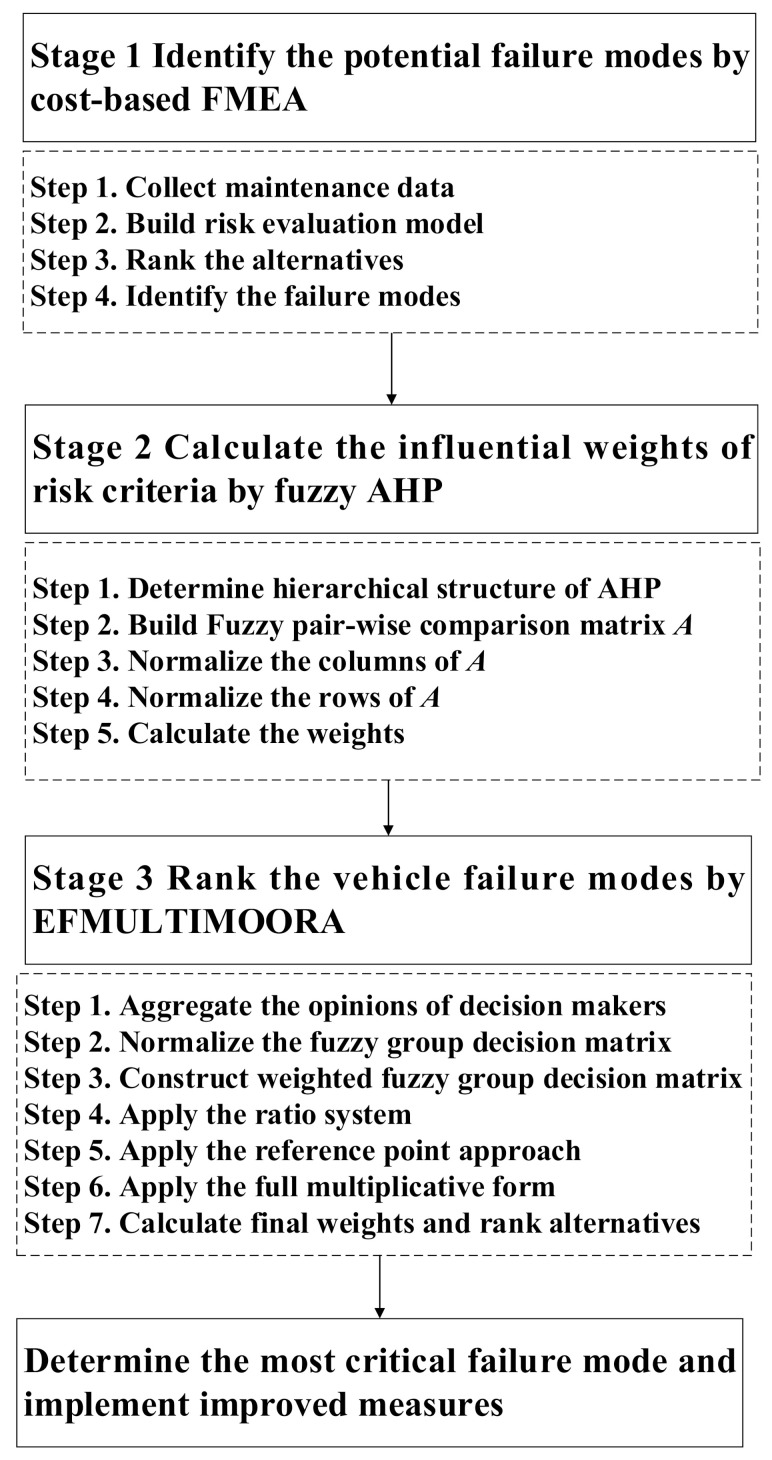
Flowchart of the proposed hybrid evaluation method.

In this section, a hybrid approach based on cost-based FMEA, FAHP, and EFMULTIMOORA is presented to address the problem of risk evaluation for vehicle failure modes with interconnections and feedback between certain risk factors. The proposed hybrid approach for evaluating the risk of vehicle failure modes consists of three main stages: (1) identify the potential failure modes using cost-based FMEA; (2) calculate the influential weights of risk criteria using FAHP; (3) rank the vehicle failure modes using EFMULTIMOORA. The flowchart of the proposed hybrid method can be seen in Figure 2.

### 3.1. Cost-Based FMEA

It is very labor intensive for experts to give their judgments on hundreds of vehicle failure modes. Thus, it is necessary to find a method to perform a preliminary screening. In the first stage, the FMEA based on the vehicle maintenance data is employed to select the top potential failure modes by calculating the value of RPNs. The steps of this modified method are stated follows:

**Step 1.** Collect maintenance data from automobile service factory.

Information is selected for target type from the factory database. The cost and probability of each failure mode can be obtained as shown in Equations (14)–(16).

(14) $$P_{ij}=\frac{m_{i}}{N}\times \frac{k_{j}}{m_{i}}=\frac{k_{j}}{N}$$

where $P_{ij}$ denotes the probability of the jth vehicle failure mode in the ith system, $m_{i}$ denotes the frequency of vehicle failure mode in the ith system, N denotes the number of vehicle samples and $k_{j}$ denotes the frequency of the jth vehicle failure mode in the ith system.

(15) $$w_{ij}\left(C\right)=w_{ij}\left(C_{t}\right)+w_{ij}\left(C_{m}\right)+w_{ij}\left(C_{0}\right)$$

where $w_{ij}\left(C\right)$, $w_{ij}\left(C_{t}\right)$, $w_{ij}\left(C_{m}\right)$, $w_{ij}\left(C_{0}\right)$ denote the total cost, man-hour cost, material cost, and the other charges of the jth vehicle failure mode in the ith system.

(16) $$w_{ij}\left(C_{t}\right)=p_{ij}\times n_{ij}$$

where $p_{ij}$ denotes the price of per maintenance man-hour of the jth failure mode in the ith system and $n_{ij}$ denotes the number of maintenance man-hours of the jth failure mode in the ith system.

**Step 2.** Build risk evaluation model based on maintenance cost.

In order to resolve the ambiguity of measuring detection difficulty and get more accurate of results, a cost-based FMEA model is proposed as shown in Equation (17) [16].

(17) $$w_{RPN}=\sum_{j=1}^{n}\left(w_{ij}\left(C\right)\times P_{ij}\right)$$

where $w_{RPN}$ denotes the value of RPN and n denotes the number of failure modes.

**Step 3**. Rank the vehicle failure modes.

In this step, the vehicle failure mode is ranked by sorting the values of $w_{RPN}$ in decreasing order.

**Step 4**. Identify the failure modes.

Select the top five failure modes to perform further risk analysis according to the results from Step 3.

### 3.2. FAHP

In the second stage, the conventional AHP is extended to calculate the weight of each criterion under a fuzzy environment. The steps of this FAHP are as follows:

**Step 1**. Build hierarchical structure to evaluate risk criteria.

**Step 2**. Construct fuzzy pair-wise comparison matrix A.

Considering Table 1, the fuzzy pair-wise comparison matrix A is determined by an expert team, which can be expressed as Equation (18).

(18) $$A={\left(a_{ij}\right)}_{n\times n},(i,j=1,2,\cdots ,n)$$

where $a_{ij}=1$ with i=j and $a_{ij}=1/a_{ji}$ with i≠j.

**Step 3**. Normalize the columns of A.

(19) $$\bar{a_{ij}}=a_{ij}/\sum_{i=1}^{n}a_{ij}\left(i,j=1,2,\cdots ,n\right)$$

**Step 4**. Normalize the rows of A.

In order to rank fuzzy numbers, the ratio of each fuzzy number with the summed values obtained in Step 3 can be computed as follows [36,37]:

(20) $$w_{i}=a_{ij}/\sum_{j=1}^{n}w_{ij}(i,j=1,2,\cdots ,n)$$

**Step 5**. Calculate ${\bar{w}}_{i}$.

The weights of risk factors are obtained by calculating the arithmetic mean value among the results obtained in Step 4, as follows.

(21) $${\bar{w}}_{i}=w_{i}/\sum_{i=1}^{n}w_{i}(i=1,2,\cdots ,n)$$

### 3.3. EFMULTIMOORA

In the final stage, the conventional MULTIMOORA method is developed under a fuzzy environment to determine the ranking priorities by calculating the final weights of each alternative in three approaches [31]. The steps of the EFMULTIMOORA are as follows:

**Step 1.** Collect the decision makers’ opinion.

The collected opinions can be recorded by the fuzzy ratings, $x_{ij}$, measured based on Table 2, which can be used to build the fuzzy group decision matrix $X={\left[x_{ij}\right]}_{m\times n}$, as follows:

(22) $$\left\{\begin{array}{l} x_{ij}=\left(x_{ij1},x_{ij2},x_{ij3}\right)\\ x_{ij1}=\frac{1}{l}\sum_{k=1}^{l}x_{ij1}^{k},x_{ij2}=\frac{1}{l}\sum_{k=1}^{l}x_{ij1}^{k},x_{ij3}=\frac{1}{l}\sum_{k=1}^{l}x_{ij1}^{k} \end{array}\right.$$

**Step 2.** Normalize X into R.

The X obtained in Step 1 can be normalized into the normalized fuzzy decision matrix $R={\left[r_{ij}\right]}_{m\times n}$, as follows.

(23) $$\begin{array}{l} r_{ij}=\left(r_{ij1},r_{ij2},r_{ij3}\right)=\left(\frac{x_{ij1}}{x_{ij3}^{*}},\frac{x_{ij2}}{x_{ij3}^{*}},\frac{x_{ij3}}{x_{ij3}^{*}}\right)\\ x_{ij3}^{*}=\sqrt{\sum_{i=1}^{m}x_{ij3}^{2}} \end{array}$$

**Step 3.** Construct weighted fuzzy group decision matrix $R^{\prime }$.

According to the weights of all risk factors obtained in the last section, the $R^{\prime }={\left[r\prime_{ij}\right]}_{m\times n}$ is formed as follows:

(24) $$r\prime_{ij}=\left(r\prime_{ij1},r\prime_{ij2},r\prime_{ij3}\right)={\bar{w}}_{j}⊗r_{ij}=\left({\bar{w}}_{j}r_{ij1},{\bar{w}}_{j}r_{ij2},{\bar{w}}_{j}r_{ij3}\right)$$

**Step 4**. Apply the ratio system.

For optimization, the responses of experts are summarized as follows [29]:

(25) $$y_{i}=({⊕}_{j=1}^{g}x_{ij}^{\prime })\Theta ({⊕}_{j=g+1}^{n}x_{ij}^{\prime })$$

where i=1,2,…,g denotes the risk factors of benefit, i=g+1,g+2,…,n denotes the risk factors of cost, and $y_{i}$ denotes the calculated assessment of vehicle failure mode $F_{i}$. Then, according to Equation (25), the weight of each risk factor with these values combined can be obtained as follows.

(26) $$w\prime_{i}=\frac{y_{i}}{\sum_{j}y_{j}}$$

**Step 5.** Apply the reference point approach.

The reference point theory is based on $R^{\prime }={\left[r\prime_{ij}\right]}_{m\times n}$, which is obtained in Step 3, whereby a fuzzy maximal objective reference point (FMORP) is also deduced [17]. Because the elements $r\prime_{ij}\in \left[0,1\right]$ and $\forall i,j\in \left[0,1\right]$, we can define this parameter as $r_{j}^{*}=(1,1,1)$ when *j* belongs to benefit criteria or $r_{j}^{*}=(0,0,0)$ when j belongs to cost criteria. Then, we can obtain the distance matrix $D={\left[d_{ij}\right]}_{m\times n}$, as follows:

(27) $$d_{ij}=d(r\prime_{ij},r_{j}^{*})=\sqrt{\frac{1}{3}\left[{\left(r\prime_{ij1}-r_{j1}^{*}\right)}^{2}+{\left(r\prime_{ij2}-r_{j2}^{*}\right)}^{2}+{\left(r\prime_{ij3}-r_{j3}^{*}\right)}^{2}\right]}$$

where $d_{ij}$ denotes the gap of vehicle failure mode $A_{i}$ in the jth risk factor $C_{j}$. The distance of each vehicle failure mode from FMORP can be measured as follows:

(28) $$d_{i}=\underset{j}{\max}\left|d_{ij}\right|$$

The weight of each risk factor in this step can be computed as follows:

(29) $$w^{\prime \prime }{}_{i}=\frac{1-d_{i}}{\sum_{j}\left(1-d_{j}\right)}$$

**Step 6**. Apply the full multiplicative form.

The utility of the ith vehicle failure with objectives to be maximized and objectives to be minimized can be express as follows:

(30) $$u_{i}=\frac{a_{i}}{b_{i}}$$

where $a_{i}={⊗}_{j=1}^{g}x^{\prime }{}_{ij}$ denotes the benefit factors, and $b_{i}={⊗}_{j=g+1}^{m}x^{\prime }{}_{ij}$ denotes the cost factors.

**Step 7**. Calculate and rank the normalized weights of each alternative.

In this step, after sorting the values $y_{i}$ and $u_{i}$ with i=1,2,…,m in decreasing order and $d_{i}$ with i=1,2,…,m in increasing order, the normalized weight of each alternative is obtained by calculating the arithmetic mean of the weights using Steps 4–6. Finally, the ranking order of all alternatives can be determined based on the normalized weight lists.

## 4. Empirical Example

In this section, in order to illustrate the feasibility and benefit of the mentioned hybrid approach, an empirical study is conducted in evaluating failure modes of a common passenger vehicle. First, we classify the critical failure from hundreds of failure modes from maintenance information using the cost-based FMEA. Second, the weight of new risk criteria, which is provided by experts, can be obtained by FAHP. Finally, the risk ranking of selected failure modes can be listed using EFMULTIMOORA. Then, a sensitivity analysis is conducted to verify the applicability of the new risk criteria. For the sake of showing the superiority of this hybrid method, the same maintenance information is also applied in other risk evaluating methods as a contrast.

### 4.1. Implementation

We selected the maintenance information for the Hyundai from an automobile service factory in Zhejiang Province, China [38]. The current investigation involved sampling and analyzing the maintenance information for 415 vehicles of the same type during March 2017, which consisted of hundreds of vehicle failure modes. The maintenance information consisted of vehicle license number, VIN number, mileage, maintenance type, failure cause, maintenance cost, etc. It involved all types of maintenance issues, such as overhaul by accidents, ordinary maintenance, and minor overhaul. This study focused on the vehicle failure modes under normal conditions, so the information belonging to overhaul by accidents was not taken into consideration. Part of the information that we collected can be seen in Table 3. According to the probability and cost of maintenance, we were able to identify the top potential vehicle failure modes by the proposed cost-based FMEA method.

Lots of quantitative and qualitative factors need to be considered when determining the top critical vehicle failure mode. It is worth noting that more vehicle failure modes were found in this application than are specified here; however, the study was limited to five critical modes for the sake of simplicity. The identified five failure modes include brake pad worn (F_1_), vehicle-mounted battery broken (F_2_), damaged or worn tires (F_3_), damaged air-conditioning filter element (F_4_), and damaged driveline bearing (F_5_) as shown in Table 4. In this study, the evaluation was conducted by a team of five decision makers, namely, DM_1_, DM_2_, DM_3_, DM_4_, and DM_5_, which involved a professional mechanical engineer, a skilled technician, a technical advisor, and two others working at the Mercedes Benz and Hyundai dealership in College Station, Texas, USA. A technical questionnaire about these failure modes was prepared concerning the evaluation criteria. The decision makers were required to provide their judgments on the interrelationship between the risk factors and the ratings for each risk factor. In addition, according to their experience and position, the propriety weights for the above-specified decision makers were 30%, 30%, 20%, 10%, and 10%, respectively, in identifying and analyzing failure modes.

The data were collected by consulting first with decision makers on their opinions. An overview of each risk factor and failure mode was provided, and each decision maker was then given a 3 × 3 linguistic scale direct-influence matrix for comparison of the three risk-evaluating criteria. The remaining portions of the matrix were left blank to be filled in by the expert team. It is worth noting that some published articles regarding vehicle failure modes were provided to the experts before filling in the questionnaire in order to assist with the accuracy of their judgments between risk factors and the performance of vehicle failure mode.

The results collected in the first and second stages are summarized in Table 5. We then utilized the proposed FAHP to calculate the weights of T, C, and B, where T, C, and B denote the required maintenance time, maintenance cost, and maintenance benefit of taking the mentioned corrective measures to decrease or eliminate the influences of vehicle failure mode, respectively. As Table 5 shows, B is the most important risk factor in this case.

Then, the normalized weight of each selected alternative was calculated using EFMULTIMOORA, considering the fuzzy linguistic terms as shown in Table 2. Table 6 shows the results for the risk criteria as provided by the five decision makers.

Finally, Table 7 presents the ranking of failure modes according to the proposed EFMULTIMOORA method. Since final weight indicates the integrated assessment of alternatives, the higher value indicates a higher weight and thus requires the most immediate attention. The ranking sequence shows that the failure mode F_1_ is the most critical choice, followed by F_2_, F_3_, F_4_, and F_5_. Consequently, utilizing the hybrid risk analysis method proposed in this study, the expert team could recommend that a worn brake pad (F_1_) is the most critical failure mode for this type of vehicle.

**Figure 3:**
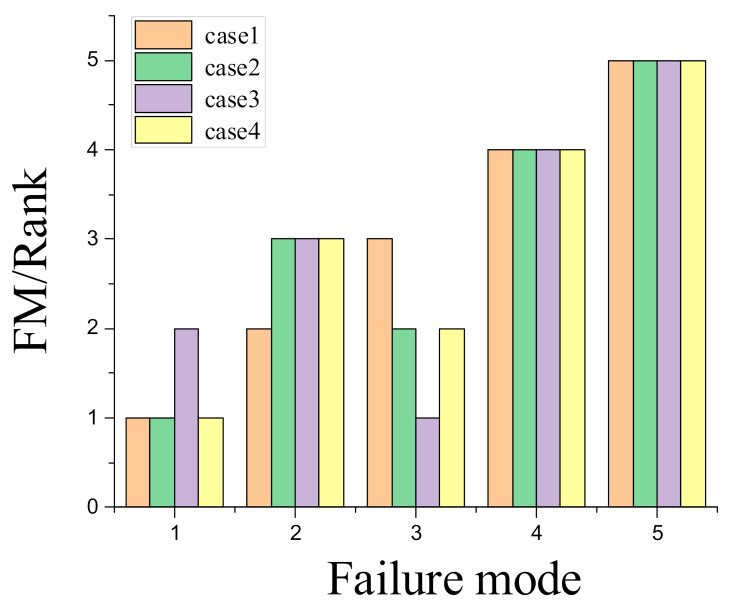
Results of ranking in sensitivity analysis.

A sensitive analysis was implemented to validate the feasibility of this hybrid approach. Thus, it was necessary to change the weights of risk factors, which were computed by the FAHP method ($w_{T}$, $w_{C}$, and $w_{B}$). As shown in Table 8, three different weights were carried out during the process of sensitive analysis. For these different cases, Case 1 came from Hyundai, and each of the other three cases had a different dominated risk factor. The results, presented in Figure 3, suggest that the weights of T, C, and B have a significant effect on risk evaluation of vehicle failure modes. Therefore, for automobile manufacturers and enterprise, it is beneficial to take both real conditions and experts’ opinions into consideration in the process of determining proper weights of risk factors and reasonable risk prioritization for vehicle failure modes. In summary, we found that this hybrid method could obtain more reasonable judgments to control risk and provide proper information for evaluating vehicle failure modes.

### 4.2. Comparisons and Discussion

The results of the above empirical study provide some interesting findings. First, when a failure mode is difficult to detect or its severity is underestimated, it can result in increased maintenance costs. Consequently, the maintenance cost can well replace the original factors of S and D. The cost-based FMEA can effectively provide a preliminary screening for hundreds of vehicle failure modes in the maintenance information. It draw experts’ attention to critical failure modes and can result in more accurate judgments.

Second, in accordance with the results of FAHP, the maintenance benefit (B) is the most important risk factor, with the effective weight of 0.387, and the maintenance time (T) is the least important one, with the effective weight of 0.283. The reason for weighing these factors in the proposed FAHP is to obtain more precise evaluation results, which can overcome the drawbacks of the conventional method effectively. Consequently, in the proposed hybrid approach, the weights of T, C, and B are applied to conduct risk evaluation for each vehicle failure mode.

Third, from the results obtained by the proposed EFMULTIMOORA, as shown in Table 7, the ranking sequence, which is F_1_ > F_2_ > F_3_ > F_4_ > F_5_, indicates that brake pad worn (F_1_) is the most critical failure mode. The reason for applying the EFMULTIMOORA method is to understand what effects the risk of a vehicle failure in the light of these three risk factors have on the whole vehicle system. Consequently, for the vehicle failure modes that take relatively little maintenance time or involve low maintenance costs and show relatively high benefits from maintenance, higher priority should be given in the optimization design for vehicles. Accordingly, automobile enterprises should perform appropriately corrective actions on these vehicle failure modes with higher priority given to them over others. Therefore, automobile enterprises can optimize vehicle design with maximum efficiency after decreasing or eliminating failure effects by taking properly corrective measures.

In addition, for validating the feasibility of the proposed hybrid evaluation approach with respect to alternatives, it is necessary to utilize the above example under comparable methods, such as cost-based FMEA, VIKOR, and MULTIMOORA, with conventional risk criteria S, O, and D. Table 9 exhibits the ranking comparisons of all vehicle failure modes as obtained by using these methods. It is clear that the rankings obtained by the proposed hybrid method are almost the same as those derived by the proposed cost-based FMEA and VIKOR methods [39] using the same risk criteria. Moreover, the ranking is also more in line with the experts’ opinions, which demonstrates the validity of the proposed hybrid method. However, this ranking order is remarkably different from that obtained by MULTIMOORA with the conventional risk criteria S, O, and D. One important reason is that the conventional risk criteria are more difficult for experts to use in expressing their real risk attitudes toward vehicle failure modes. Each industry should establish its own risk evaluation system. This cognitive error, coming from the difference of fields, may increase the difficulty in making decisions, which may cause inconsistencies in risk evaluation. The comparisons demonstrate the feasibility and applicability of the proposed hybrid method for evaluating the risk of vehicle failure modes. Compared with previous approaches on evaluating vehicle failure modes, this hybrid method has the following advantages.

First, the proposed method selects the top five critical failure modes from maintenance information, which makes the results consistent with the real vehicle conditions. In addition, it is beneficial for the experts to make judgments on the critical alternatives after using this preliminary screening method.

Second, the risk criteria of failure modes and their interdependent relationships are judged using fuzzy numbers rather than crisp values. This allows for more reasonable and precise expression of their risk attitudes during the process of evaluation. In addition, the new risk criteria of T, C, and B are easy to understand for experts, which enable them to express their judgement more accurately.

Third, the priority ranking of failure modes is determined by EFMULTIMOORA, which can be easily carried out with high applicability. Therefore, the proposed risk-evaluating framework can result in a highly robust evaluation of the vehicle failure modes and can have great feasibility in real-world applications.

## 5. Conclusions

In this paper, we propose a three-stage scheme to evaluate the risk of vehicle failure modes. In order to present the feasibility of our hybrid method, an empirical study was conducted for a common vehicle. By utilizing the method of FAHP, the criteria for evaluating failure modes were proven to be interrelated and interdependent. Considering the effects of all factors, priority should be given to the most beneficial factors when making decisions. In this hybrid method, the ranking of vehicle failure modes is presented by the suggested EFMULTIMOORA with the new risk criteria of T, C, and B. As a result, the alternative F_1_ is the most critical failure mode for the Hyundai vehicle, followed by the alternatives F_2_, F_3_, F_4_, and F_5_. The selected ranking is similar to those obtained by cost-based FMEA and VIKOR methods. Therefore, it has been validated that the proposed fuzzy hybrid method is an effective tool in evaluating the risk of vehicle failure modes with interdependent factors and compromise alternatives.

In addition, we point out several open issues for future research. First, our proposed hybrid method is restricted to the failure mode evaluation for Hyundai; it can be employed with similar reliability for other types of vehicles in future research. Furthermore, to further validate the reliability of the proposed method, extensive research should be conducted in different industries in the future.

## Figures and Tables

**Table 1:** Linguistic variables for rating the risk factors.

Linguistic Variable	Triangular Fuzzy Number
Equal	(1,1,1)
Moderate	(1,1,1.5)
Strong	(1,1.5,2)
Very strong	(1.5,2,2.5)
Extreme	(2,2.5,3)

**Table 2:** Linguistic variables for rating the vehicle failure modes.

Linguistic Variable	Triangular Fuzzy Number
Very low (VL)	(0,0,1)
Low (L)	(1,2,3)
Medium low (ML)	(1,3,5)
Medium (M)	(3,5,7)
Medium high (MH)	(5,7,9)
High (H)	(7,9,10)
Very high (VH)	(9,10,10)

**Table 3:** Part of the maintenance information for the Hyundai vehicles.

No.	VIN	Mileage	Type	Failure Cause	Cost
1	LBELMBKC3DY322169	9200 km	Minor overhaul	Replace oil and air filter	¥120
2	LBELMBKC9EY572517	12,560 km	Minor overhaul	Replace trunk switch	¥180
3	LBELMBKC0GY705216	3912 km	First maintenance	None	¥0

**Table 4:** Part of the results of evaluation.

Failure Systems	Failure Mode	Probability	Cost/¥	RPN
Engine	Replace oil and air filter	0.0048	120	0.576
	Replace spark plug	0.0072	180	1.296
Drive system	Replace the fixed card buckle	0.0072	60	0.432
	Replace the bearing	0.0096	200	1.92
Steering system	Replacement tire	0.0048	800	3.84
Traveling system	Check steering wheel	0.012	60	0.72
Barking system	Replace brake block	0.024	185	4.44
Instrument lighting	Equipped with low LED	0.0024	600	1.44
	Maintain high beam	0.0024	400	0.72
Safety device	Adjust the gap round the auto door	0.0024	600	1.44
Air-conditioning system	Replace air filter	0.038	60	2.28
Electronic equipment	Replace trunk switch	0.0024	180	0.432
	Replace HBC-A	0.014	20	0.28
	Replace battery	0.0096	400	3.84
Bodywork	polishing	0.0048	380	1.824

**Table 5:** Fuzzy pair-wise comparison matrix for the three risk factors.

Risk Criteria	T	C	B	Weight
T	(1,1,1)	(2/3,1,1)	(1/2,2/3,1)	0.283
C	(1,1,1.5)	(1,1,1)	(2/3,1,1)	0.330
B	(1,1.5,2)	(1,1,1.5)	(1,1,1)	0.387

T, C, B denote the maintenance time, maintenance cost, and maintenance benefit of failure modes.

**Table 6:** The results of the three risk factors for vehicle failure modes.

Criteria	DMs	Weight	F_1_	F_2_	F_3_	F_4_	F_5_
T (−)	DM_1_	20%	M	M	M	M	H
	DM_2_	30%	VL	VL	VL	VL	MH
	DM_3_	30%	VL	ML	VL	VL	VH
	DM_4_	10%	ML	VL	VL	VL	ML
	DM_5_	10%	L	L	L	VL	H
C (−)	DM_1_	20%	ML	M	M	M	MH
	DM_2_	30%	ML	ML	MH	L	H
	DM_3_	30%	MH	ML	MH	VL	VH
	DM_4_	10%	L	L	VL	L	L
	DM_5_	10%	M	ML	L	VL	VH
B (+)	DM_1_	20%	VH	VH	VH	MH	MH
	DM_2_	30%	H	VH	VH	M	H
	DM_3_	30%	VH	H	VH	M	VH
	DM_4_	10%	H	MH	VH	M	VH
	DM_5_	10%	VH	MH	VH	MH	VH

T, C, B denote the maintenance time, maintenance cost, and maintenance benefit of failure modes.

**Table 7:** The ranking alternatives according to extended fuzzy multi-objective optimization by ratio analysis plus full multiplicative form (EFMULTIMOORA).

FM	y_i_	Rank	d_i_	Rank	u_i_	Rank	Weight	Rank
F_1_	(−0.0654, 0.0121, 0.0738)	1	0.8310	5	(14.38, 45.30, 158.5)	3	0.2674	1
F_2_	(−0.0344, 0.0356, 0.0805)	2	0.8390	2	(12.36, 39.97, 213.1)	2	0.2213	2
F_3_	(−0.0449, 0.0277, 0.0614)	4	0.8237	4	(15.85, 46.97, 117.5)	4	0.2188	3
F_4_	(0.0124, 0.0348, 0.0271)	3	0.8983	1	(16.60, 94.69, 385.5)	1	0.2186	4
F_5_	(−0.0705, −0.0362, 0.0555)	5	0.8390	2	(2.763, 4.120, 6.854)	5	0.0736	5

FM denotes Failure mode; y_i_, d_i_, u_i_, denote the risk criteria.

**Table 8:** The percentages of sensitivity analysis cases.

Criteria	Case 1	Case 2	Case 3	Case 4
T	0.283	0.1	0.1	0.6
C	0.330	0.2	0.8	0.3
B	0.387	0.7	0.1	0.1

**Table 9:** The results of comparisons.

Failure Mode	Cost-Based FMEA		VIKOR with T, C, B				MULTIMOORA with S, O, D			
	RPN	Rank	S	R	Q	Rank	y_i_	d_i_	u_i_	Rank
F_1_	4.44	1	1.8229	1.1321	0.6791	2	0.4990	0.9115	0.0329	1
F_2_	3.84	2	1.9322	0.6838	0.0720	1	0.2840	0.9256	0.0074	4
F_3_	3.84	2	1.9062	1.5709	1.3990	3	0.4831	0.9205	0.0281	3
F_4_	2.28	4	3.0868	2.7551	3.9718	4	0.2045	0.9538	0.0032	5
F_5_	1.92	5	5.9008	2.7802	5.8657	5	0.4841	0.9115	0.308	2

S, Q, R are the risk criteria in VIKOR method.

## Data Availability

Data sharing not applicable.
